# Supplementary material for: Adherence of Trichomonas vaginalis to SiHa Cells is Inhibited by Diphenyleneiodonium
Source: Microorganisms. 2020 Oct 13;8(10):1570. doi: 10.3390/microorganisms8101570 (PMC7600062; doi:10.3390/microorganisms8101570)
Supplement: Supplementary file 1 [file microorganisms-08-01570-s001.pdf]

Figure S1

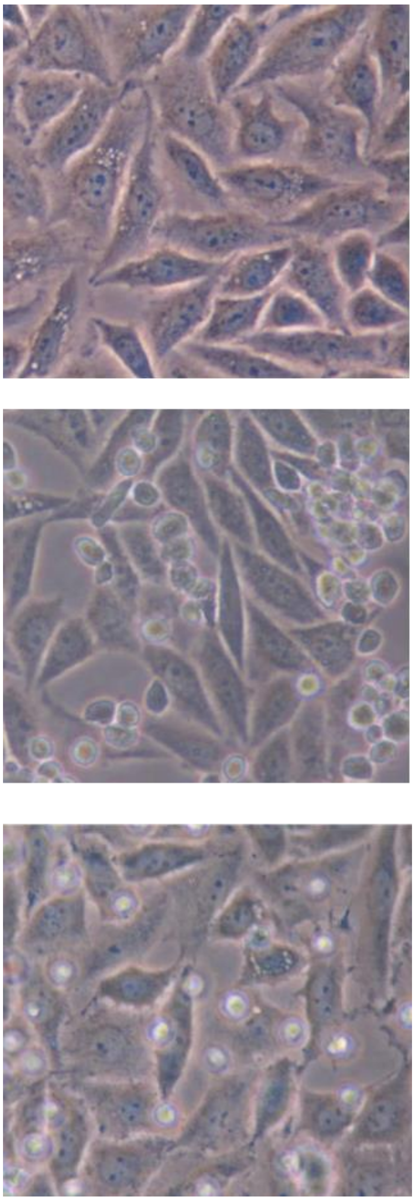

Figure S1. Representative microscopic images of SiHa and *T.v* mixed cultures with or without DPI (1 μM) treatment

Figure S2

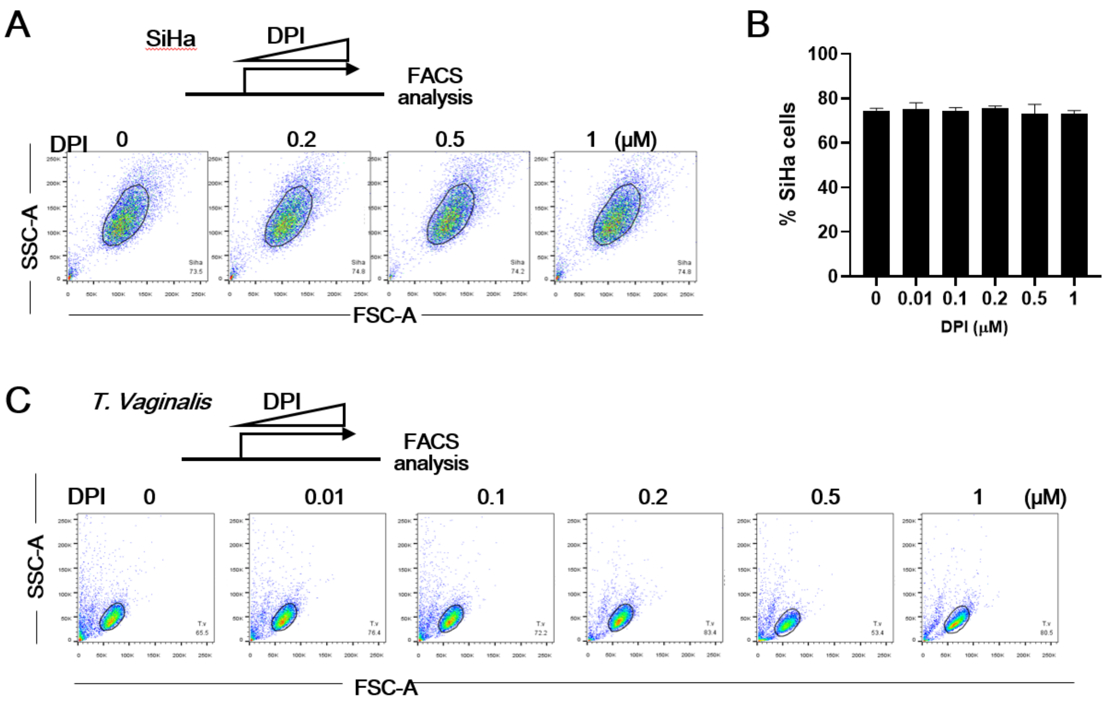

Figure S2. DPI treatment on SiHa cells or *T. vaginalis* alone did not affect cell viability. (A) SiHa cells were treated with various concentration of DPI for 2 h and analyzed by flow cytometry. (B) SiHa cell viability quantification (n=3). (C) *T. vaginalis* culture was treated with various concentration of DPI for 2 h and analyzed by flow cytometry. Mean ± SD, one-way ANOVA.
